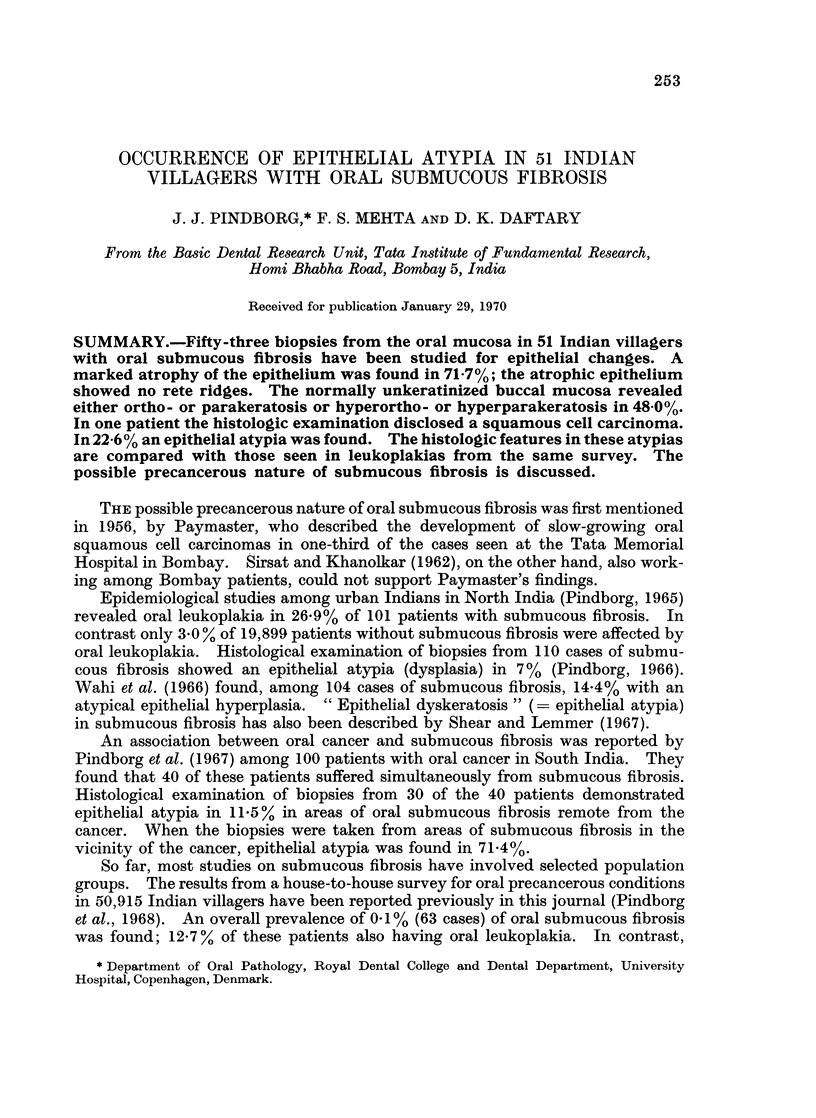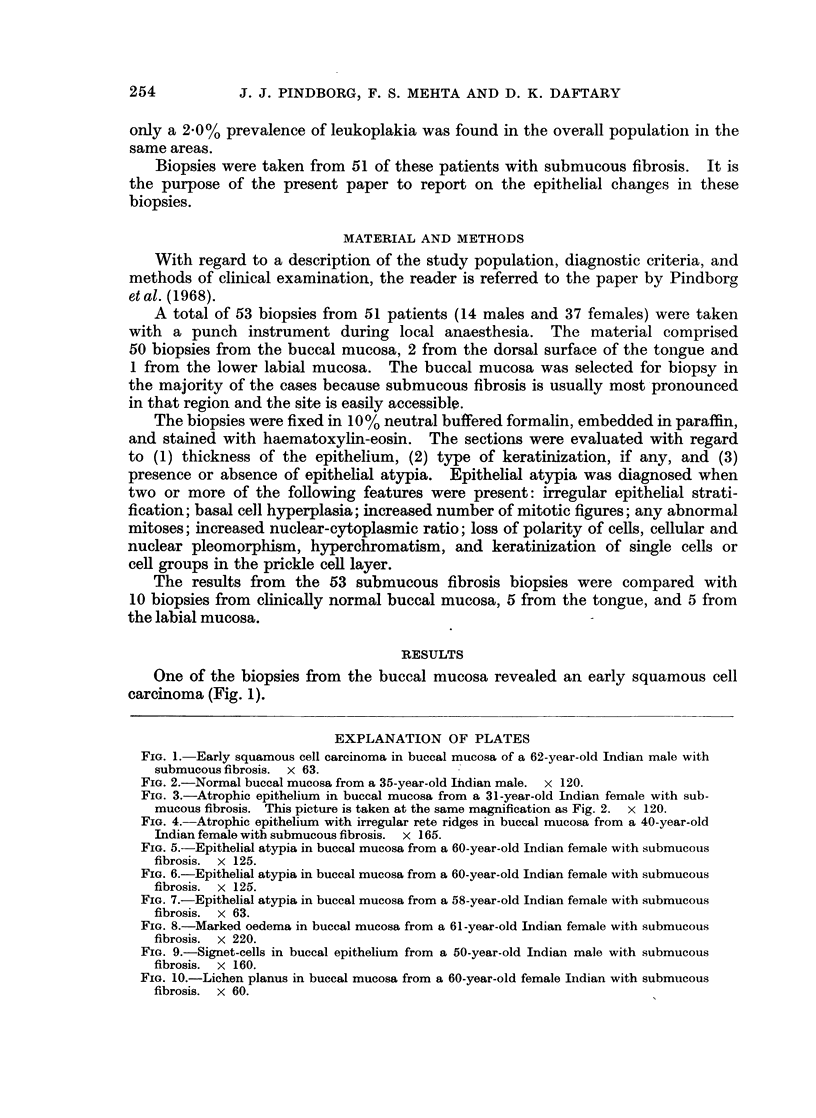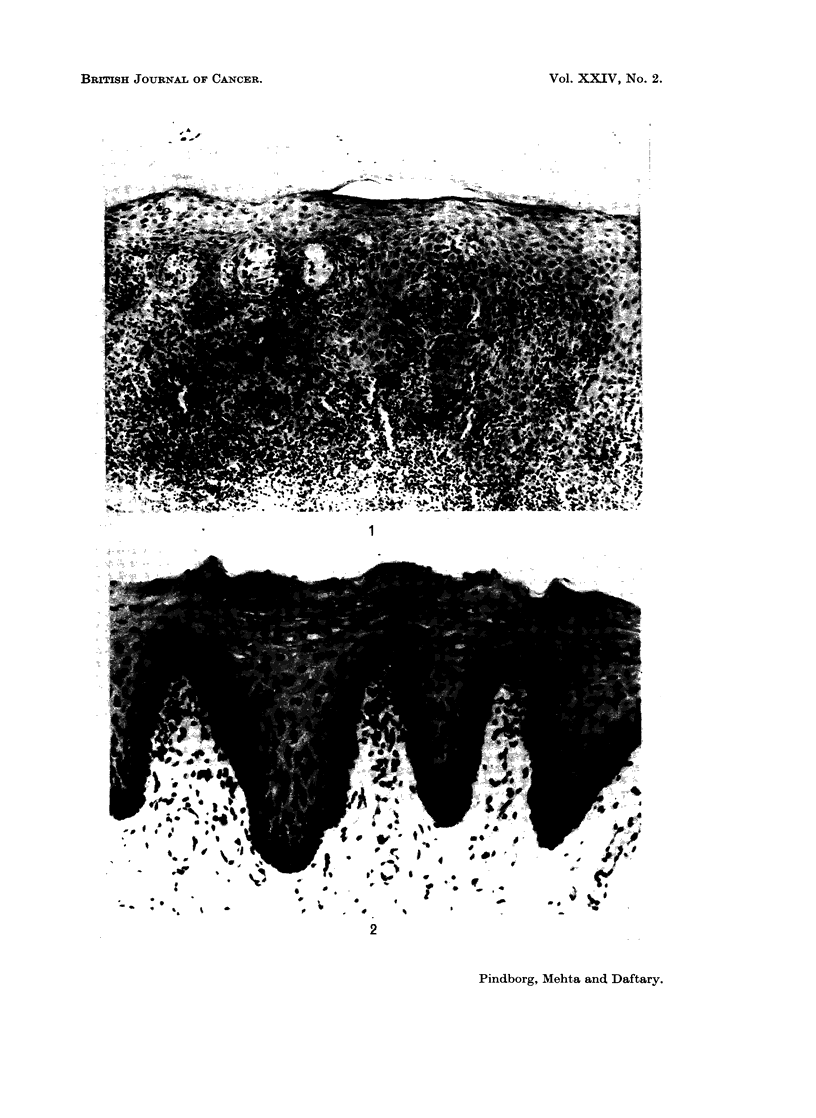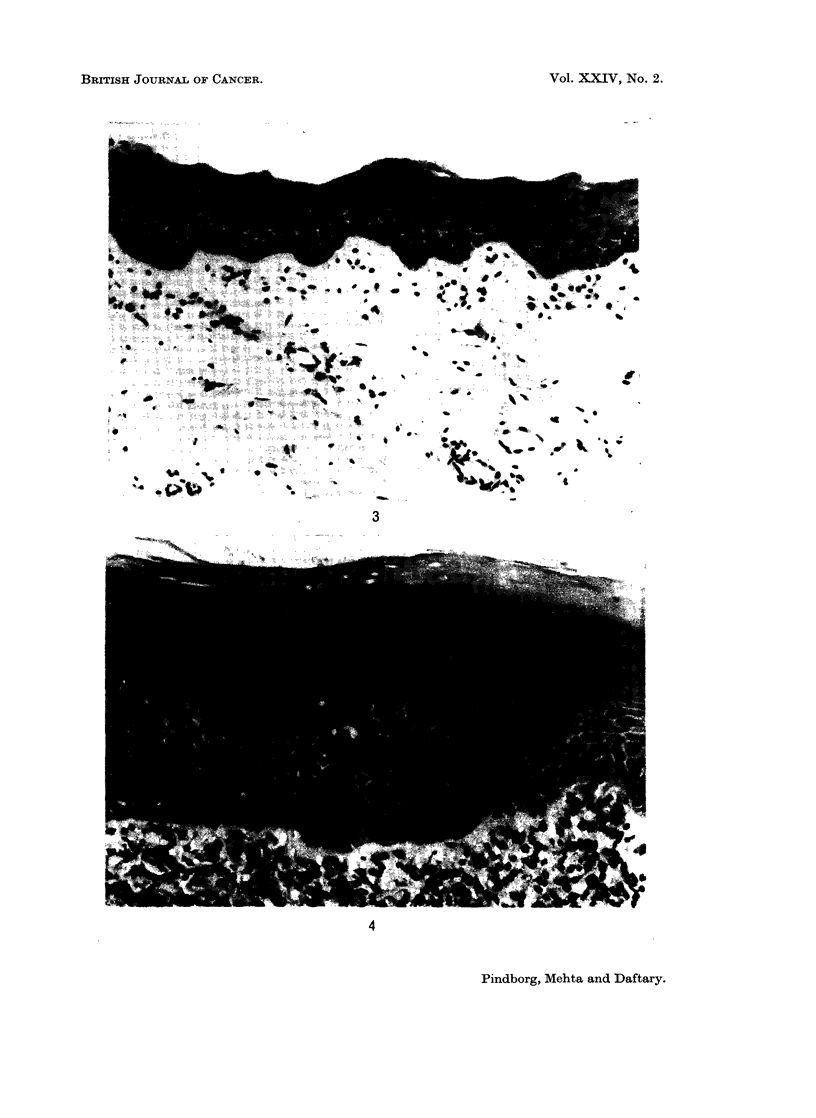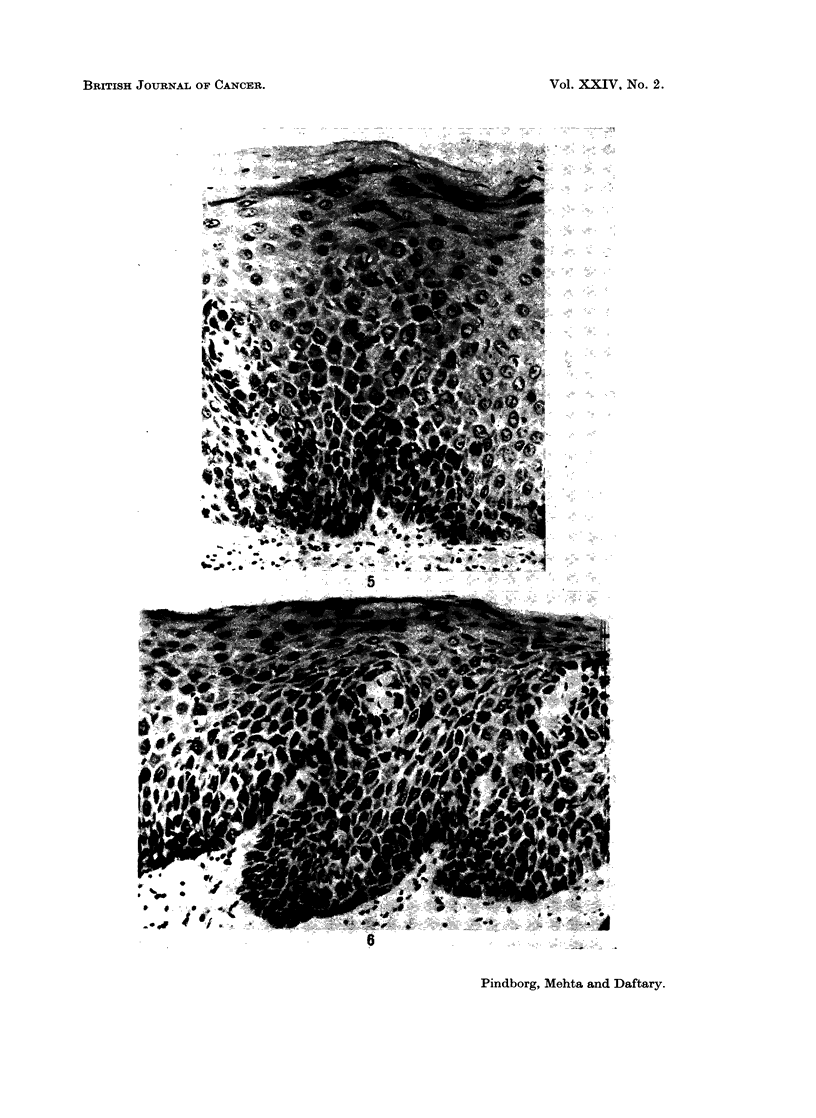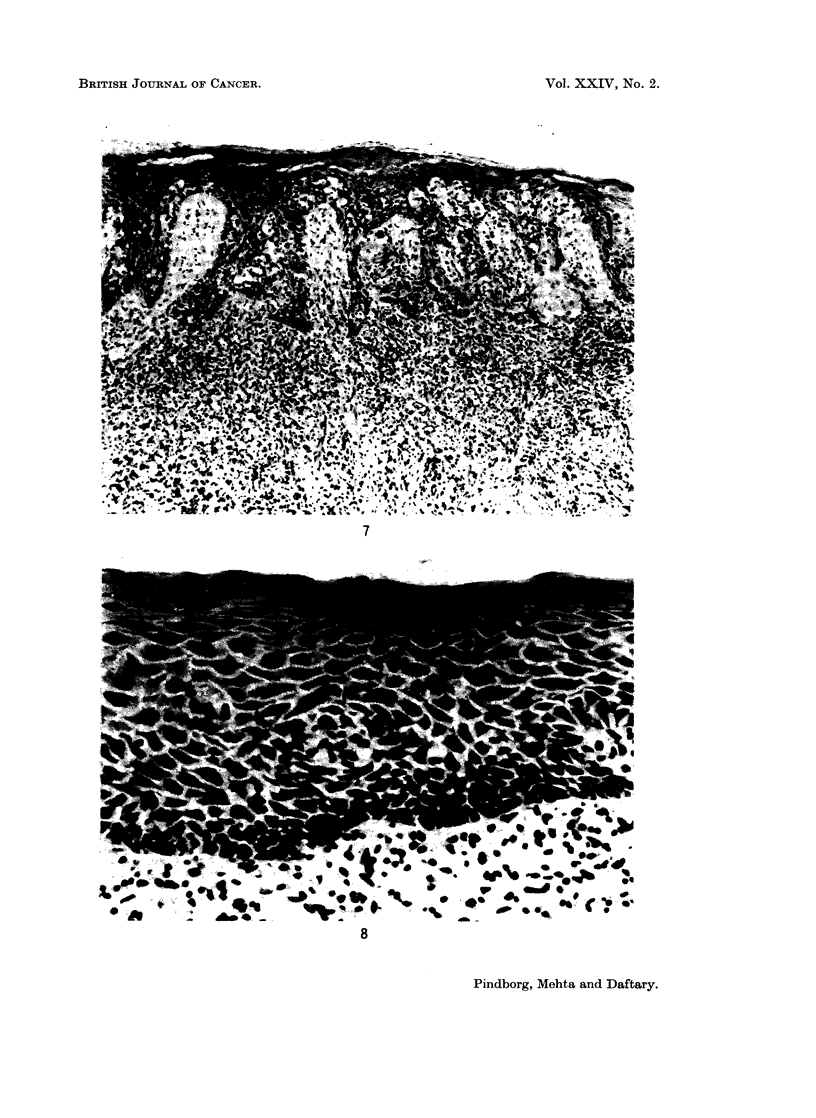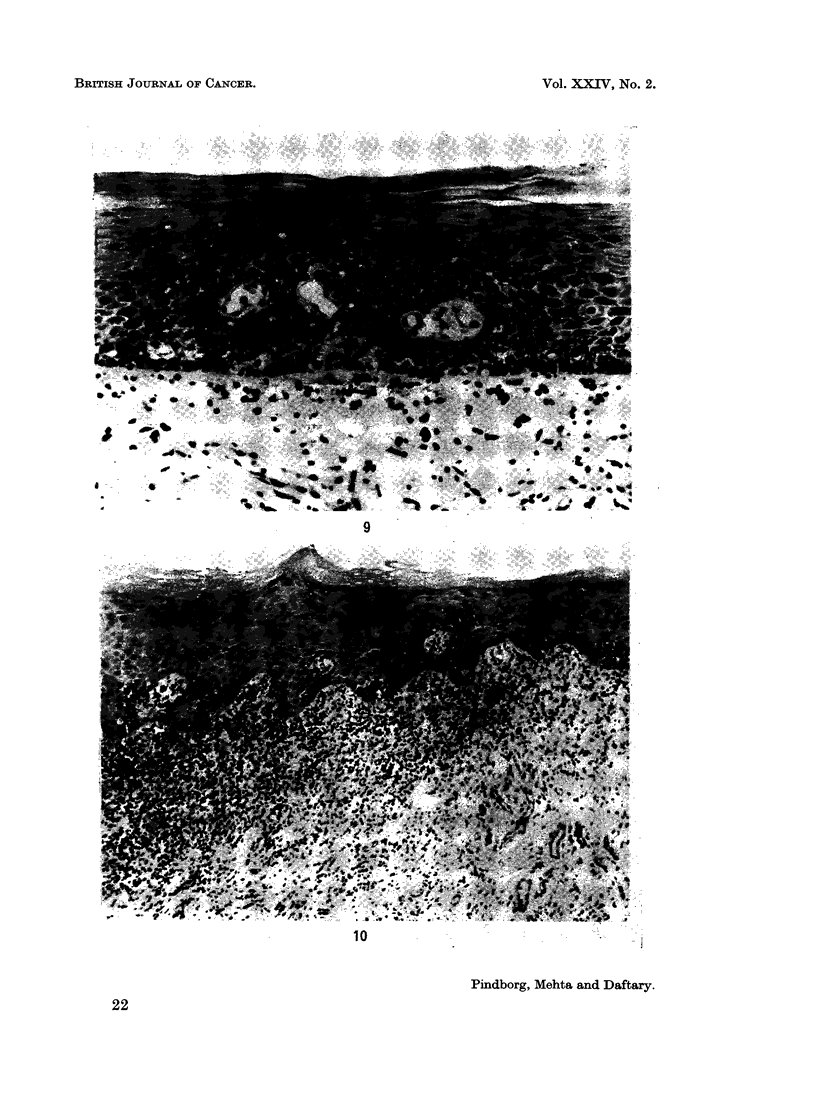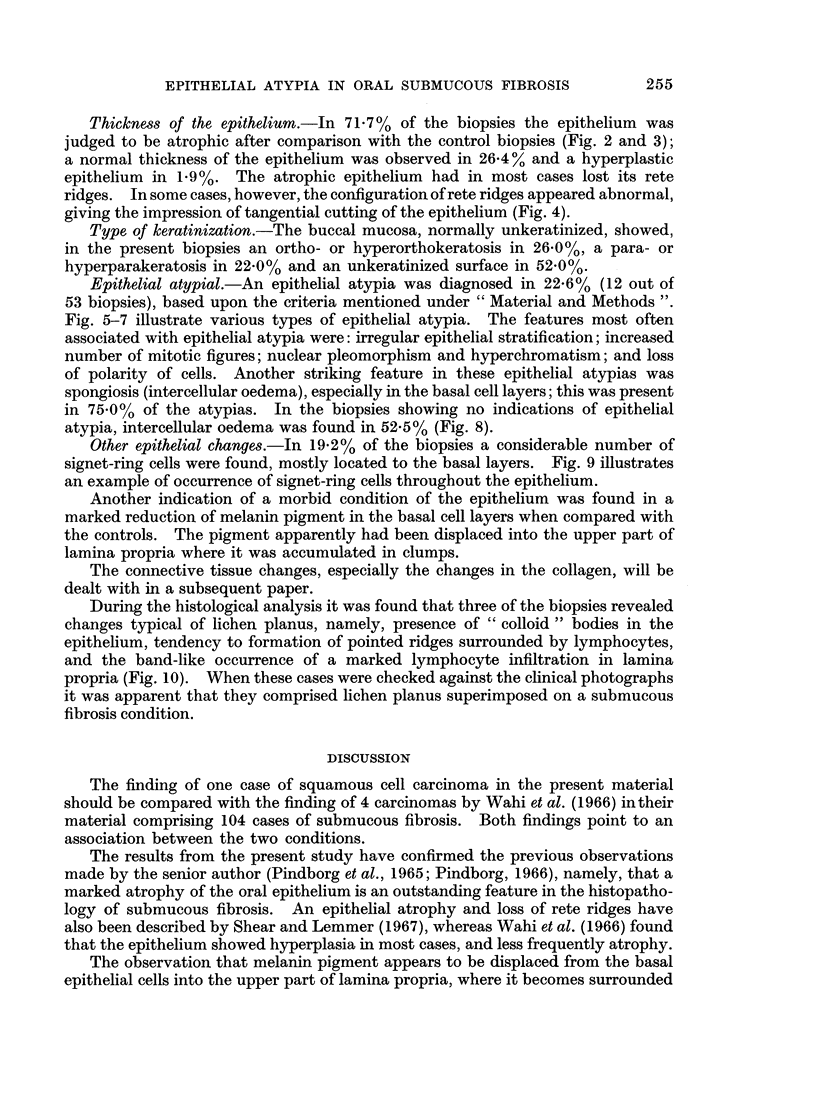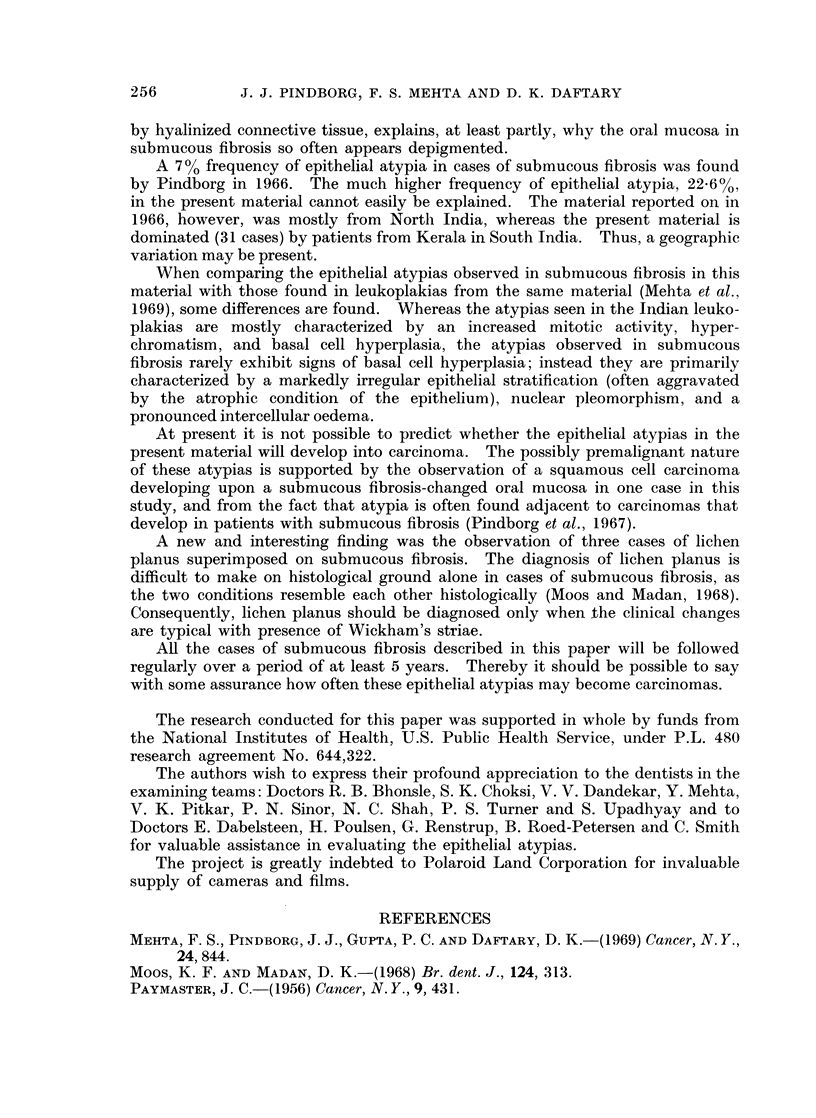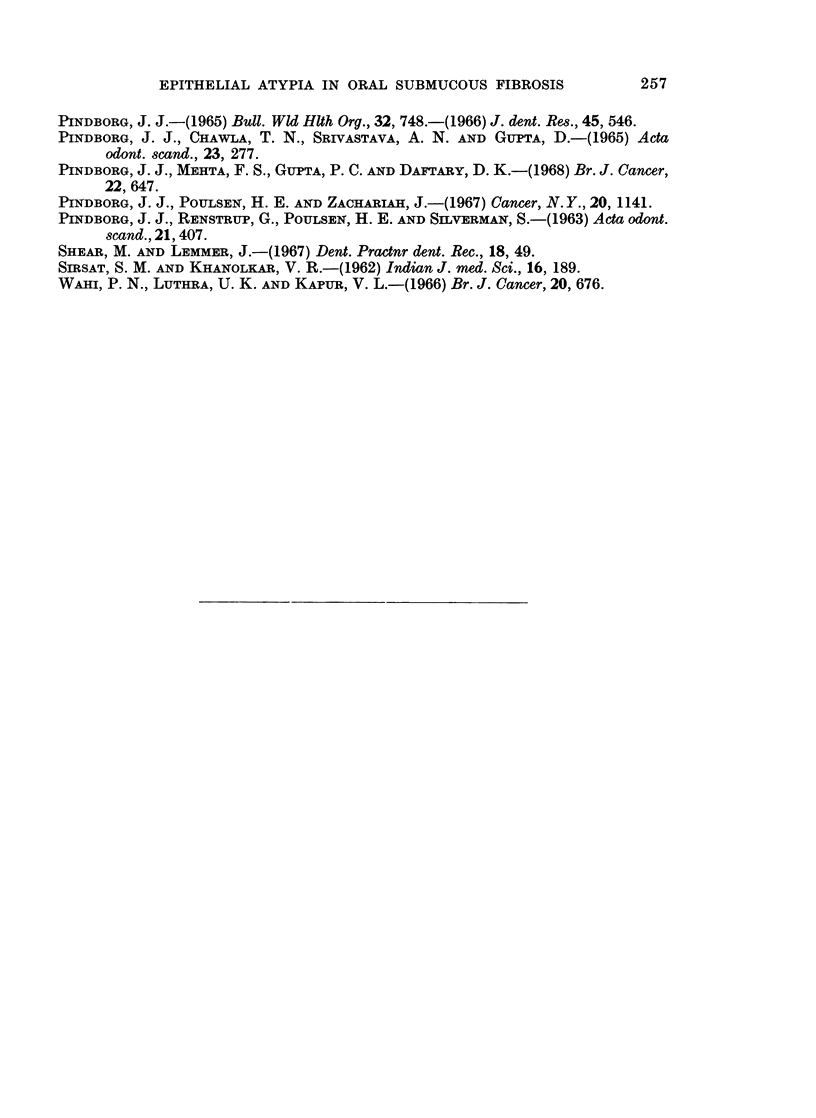# Occurrence of Epithelial Atypia in 51 Indian Villagers with Oral Submucous Fibrosis

**DOI:** 10.1038/bjc.1970.28

**Published:** 1970-06

**Authors:** J. J. Pindborg, F. S. Mehta, D. K. Daftary

## Abstract

**Images:**


### OCCURRENCE OF EPITHELIAL ATYPIA IN 51 INDIAN VILLAGERS WITH ORAL SUBMUCOUS FIBROSIS

J. J. PINDBORG,* F. S. MEHTA AND D. K. DAFTARY

*From the Basic Dental Research Unit, Tata Institute of Fundamental Research, Homi Bhabha Road, Bombay 5, India*

Received for publication January 29, 1970

**SUMMARY.—Fifty-three biopsies from the oral mucosa in 51 Indian villagers with oral submucous fibrosis have been studied for epithelial changes. A marked atrophy of the epithelium was found in 71·7%; the atrophic epithelium showed no rete ridges. The normally unkeratinized buccal mucosa revealed either ortho- or parakeratosis or hyperortho- or hyperparakeratosis in 48·0%. In one patient the histologic examination disclosed a squamous cell carcinoma. In 22·6% an epithelial atypia was found. The histologic features in these atypias are compared with those seen in leukoplakias from the same survey. The possible precancerous nature of submucous fibrosis is discussed.**

THE possible precancerous nature of oral submucous fibrosis was first mentioned in 1956, by Paymaster, who described the development of slow-growing oral squamous cell carcinomas in one-third of the cases seen at the Tata Memorial Hospital in Bombay. Sirsat and Khanolkar (1962), on the other hand, also working among Bombay patients, could not support Paymaster's findings.

Epidemiological studies among urban Indians in North India (Pindborg, 1965) revealed oral leukoplakia in 26·9% of 101 patients with submucous fibrosis. In contrast only 3·0% of 19,899 patients without submucous fibrosis were affected by oral leukoplakia. Histological examination of biopsies from 110 cases of submucous fibrosis showed an epithelial atypia (dysplasia) in 7% (Pindborg, 1966). Wahi *et al.* (1966) found, among 104 cases of submucous fibrosis, 14·4% with an atypical epithelial hyperplasia. " Epithelial dyskeratosis " (= epithelial atypia) in submucous fibrosis has also been described by Shear and Lemmer (1967).

An association between oral cancer and submucous fibrosis was reported by Pindborg *et al.* (1967) among 100 patients with oral cancer in South India. They found that 40 of these patients suffered simultaneously from submucous fibrosis. Histological examination of biopsies from 30 of the 40 patients demonstrated epithelial atypia in 11·5% in areas of oral submucous fibrosis remote from the cancer. When the biopsies were taken from areas of submucous fibrosis in the vicinity of the cancer, epithelial atypia was found in 71·4%.

So far, most studies on submucous fibrosis have involved selected population groups. The results from a house-to-house survey for oral precancerous conditions in 50,915 Indian villagers have been reported previously in this journal (Pindborg *et al.*, 1968). An overall prevalence of 0·1% (63 cases) of oral submucous fibrosis was found; 12·7% of these patients also having oral leukoplakia. In contrast,

* Department of Oral Pathology, Royal Dental College and Dental Department, University Hospital, Copenhagen, Denmark.

only a 2·0% prevalence of leukoplakia was found in the overall population in the same areas.

Biopsies were taken from 51 of these patients with submucous fibrosis. It is the purpose of the present paper to report on the epithelial changes in these biopsies.

### MATERIAL AND METHODS

With regard to a description of the study population, diagnostic criteria, and methods of clinical examination, the reader is referred to the paper by Pindborg *et al.* (1968).

A total of 53 biopsies from 51 patients (14 males and 37 females) were taken with a punch instrument during local anaesthesia. The material comprised 50 biopsies from the buccal mucosa, 2 from the dorsal surface of the tongue and 1 from the lower labial mucosa. The buccal mucosa was selected for biopsy in the majority of the cases because submucous fibrosis is usually most pronounced in that region and the site is easily accessible.

The biopsies were fixed in 10% neutral buffered formalin, embedded in paraffin, and stained with haematoxylin-eosin. The sections were evaluated with regard to (1) thickness of the epithelium, (2) type of keratinization, if any, and (3) presence or absence of epithelial atypia. Epithelial atypia was diagnosed when two or more of the following features were present: irregular epithelial stratification; basal cell hyperplasia; increased number of mitotic figures; any abnormal mitoses; increased nuclear-cytoplasmic ratio; loss of polarity of cells, cellular and nuclear pleomorphism, hyperchromatism, and keratinization of single cells or cell groups in the prickle cell layer.

The results from the 53 submucous fibrosis biopsies were compared with 10 biopsies from clinically normal buccal mucosa, 5 from the tongue, and 5 from the labial mucosa.

### RESULTS

One of the biopsies from the buccal mucosa revealed an early squamous cell carcinoma (Fig. 1).

---

### EXPLANATION OF PLATES

FIG. 1.—Early squamous cell carcinoma in buccal mucosa of a 62-year-old Indian male with submucous fibrosis. × 63.

FIG. 2.—Normal buccal mucosa from a 35-year-old Indian male. × 120.

FIG. 3.—Atrophic epithelium in buccal mucosa from a 31-year-old Indian female with submucous fibrosis. This picture is taken at the same magnification as Fig. 2. × 120.

FIG. 4.—Atrophic epithelium with irregular rete ridges in buccal mucosa from a 40-year-old Indian female with submucous fibrosis. × 165.

FIG. 5.—Epithelial atypia in buccal mucosa from a 60-year-old Indian female with submucous fibrosis. × 125.

FIG. 6.—Epithelial atypia in buccal mucosa from a 60-year-old Indian female with submucous fibrosis. × 125.

FIG. 7.—Epithelial atypia in buccal mucosa from a 58-year-old Indian female with submucous fibrosis. × 63.

FIG. 8.—Marked oedema in buccal mucosa from a 61-year-old Indian female with submucous fibrosis. × 220.

FIG. 9.—Signet-cells in buccal epithelium from a 50-year-old Indian male with submucous fibrosis. × 160.

FIG. 10.—Lichen planus in buccal mucosa from a 60-year-old female Indian with submucous fibrosis. × 60.

1

2

Pindborg, Mehta and Daftary.

3

4

Pindborg, Mehta and Daftary.

5

6

Pindborg, Mehta and Daftary.

7

8

Pindborg, Mehta and Daftary.

9

10

Pindborg, Mehta and Daftary.

*Thickness of the epithelium.*—In 71·7% of the biopsies the epithelium was judged to be atrophic after comparison with the control biopsies (Fig. 2 and 3); a normal thickness of the epithelium was observed in 26·4% and a hyperplastic epithelium in 1·9%. The atrophic epithelium had in most cases lost its rete ridges. In some cases, however, the configuration of rete ridges appeared abnormal, giving the impression of tangential cutting of the epithelium (Fig. 4).

*Type of keratinization.*—The buccal mucosa, normally unkeratinized, showed, in the present biopsies an ortho- or hyperorthokeratosis in 26·0%, a para- or hyperparakeratosis in 22·0% and an unkeratinized surface in 52·0%.

*Epithelial atypial.*—An epithelial atypia was diagnosed in 22·6% (12 out of 53 biopsies), based upon the criteria mentioned under " Material and Methods ". Fig. 5–7 illustrate various types of epithelial atypia. The features most often associated with epithelial atypia were: irregular epithelial stratification; increased number of mitotic figures; nuclear pleomorphism and hyperchromatism; and loss of polarity of cells. Another striking feature in these epithelial atypias was spongiosis (intercellular oedema), especially in the basal cell layers; this was present in 75·0% of the atypias. In the biopsies showing no indications of epithelial atypia, intercellular oedema was found in 52·5% (Fig. 8).

*Other epithelial changes.*—In 19·2% of the biopsies a considerable number of signet-ring cells were found, mostly located to the basal layers. Fig. 9 illustrates an example of occurrence of signet-ring cells throughout the epithelium.

Another indication of a morbid condition of the epithelium was found in a marked reduction of melanin pigment in the basal cell layers when compared with the controls. The pigment apparently had been displaced into the upper part of lamina propria where it was accumulated in clumps.

The connective tissue changes, especially the changes in the collagen, will be dealt with in a subsequent paper.

During the histological analysis it was found that three of the biopsies revealed changes typical of lichen planus, namely, presence of " colloid " bodies in the epithelium, tendency to formation of pointed ridges surrounded by lymphocytes, and the band-like occurrence of a marked lymphocyte infiltration in lamina propria (Fig. 10). When these cases were checked against the clinical photographs it was apparent that they comprised lichen planus superimposed on a submucous fibrosis condition.

### DISCUSSION

The finding of one case of squamous cell carcinoma in the present material should be compared with the finding of 4 carcinomas by Wahi *et al.* (1966) in their material comprising 104 cases of submucous fibrosis. Both findings point to an association between the two conditions.

The results from the present study have confirmed the previous observations made by the senior author (Pindborg *et al.*, 1965; Pindborg, 1966), namely, that a marked atrophy of the oral epithelium is an outstanding feature in the histopathology of submucous fibrosis. An epithelial atrophy and loss of rete ridges have also been described by Shear and Lemmer (1967), whereas Wahi *et al.* (1966) found that the epithelium showed hyperplasia in most cases, and less frequently atrophy.

The observation that melanin pigment appears to be displaced from the basal epithelial cells into the upper part of lamina propria, where it becomes surrounded by hyalinized connective tissue, explains, at least partly, why the oral mucosa in submucous fibrosis so often appears depigmented.

A 7% frequency of epithelial atypia in cases of submucous fibrosis was found by Pindborg in 1966. The much higher frequency of epithelial atypia, 22·6%, in the present material cannot easily be explained. The material reported on in 1966, however, was mostly from North India, whereas the present material is dominated (31 cases) by patients from Kerala in South India. Thus, a geographic variation may be present.

When comparing the epithelial atypias observed in submucous fibrosis in this material with those found in leukoplakias from the same material (Mehta *et al.*, 1969), some differences are found. Whereas the atypias seen in the Indian leukoplakias are mostly characterized by an increased mitotic activity, hyperchromatism, and basal cell hyperplasia, the atypias observed in submucous fibrosis rarely exhibit signs of basal cell hyperplasia; instead they are primarily characterized by a markedly irregular epithelial stratification (often aggravated by the atrophic condition of the epithelium), nuclear pleomorphism, and a pronounced intercellular oedema.

At present it is not possible to predict whether the epithelial atypias in the present material will develop into carcinoma. The possibly premalignant nature of these atypias is supported by the observation of a squamous cell carcinoma developing upon a submucous fibrosis-changed oral mucosa in one case in this study, and from the fact that atypia is often found adjacent to carcinomas that develop in patients with submucous fibrosis (Pindborg *et al.*, 1967).

A new and interesting finding was the observation of three cases of lichen planus superimposed on submucous fibrosis. The diagnosis of lichen planus is difficult to make on histological ground alone in cases of submucous fibrosis, as the two conditions resemble each other histologically (Moos and Madan, 1968). Consequently, lichen planus should be diagnosed only when the clinical changes are typical with presence of Wickham's striae.

All the cases of submucous fibrosis described in this paper will be followed regularly over a period of at least 5 years. Thereby it should be possible to say with some assurance how often these epithelial atypias may become carcinomas.

The research conducted for this paper was supported in whole by funds from the National Institutes of Health, U.S. Public Health Service, under P.L. 480 research agreement No. 644,322.

The authors wish to express their profound appreciation to the dentists in the examining teams: Doctors R. B. Bhonsle, S. K. Choksi, V. V. Dandekar, Y. Mehta, V. K. Pitkar, P. N. Sinor, N. C. Shah, P. S. Turner and S. Upadhyay and to Doctors E. Dabelsteen, H. Poulsen, G. Renstrup, B. Roed-Petersen and C. Smith for valuable assistance in evaluating the epithelial atypias.

The project is greatly indebted to Polaroid Land Corporation for invaluable supply of cameras and films.

### REFERENCES

MEHTA, F. S., PINDBORG, J. J., GUPTA, P. C. AND DAFTARY, D. K.—(1969) *Cancer, N.Y.*, **24**, 844.

MOOS, K. F. AND MADAN, D. K.—(1968) *Br. dent. J.*, **124**, 313.

PAYMASTER, J. C.—(1956) *Cancer, N.Y.*, **9**, 431.

PINDBORG, J. J.—(1965) *Bull. Wld Hlth Org.*, **32**, 748.—(1966) *J. dent. Res.*, **45**, 546.
PINDBORG, J. J., CHAWLA, T. N., SRIVASTAVA, A. N. AND GUPTA, D.—(1965) *Acta odont. scand.*, **23**, 277.
PINDBORG, J. J., MEHTA, F. S., GUPTA, P. C. AND DAFTARY, D. K.—(1968) *Br. J. Cancer*, **22**, 647.
PINDBORG, J. J., POULSEN, H. E. AND ZACHARIAH, J.—(1967) *Cancer, N.Y.*, **20**, 1141.
PINDBORG, J. J., RENSTRUP, G., POULSEN, H. E. AND SILVERMAN, S.—(1963) *Acta odont. scand.*, **21**, 407.
SHEAR, M. AND LEMMER, J.—(1967) *Dent. Practnr dent. Rec.*, **18**, 49.
SIRSAT, S. M. AND KHANOLKAR, V. R.—(1962) *Indian J. med. Sci.*, **16**, 189.
WAHI, P. N., LUTHRA, U. K. AND KAPUR, V. L.—(1966) *Br. J. Cancer*, **20**, 676.